# Global Dietary and Herbal Supplement Use during COVID-19—A Scoping Review

**DOI:** 10.3390/nu15030771

**Published:** 2023-02-02

**Authors:** Ishaan Arora, Shecoya White, Rahel Mathews

**Affiliations:** Department of Food Science, Nutrition and Health Promotion, Mississippi State University, Starkville, MS 39762, USA

**Keywords:** COVID-19, lockdown, supplements, dietary supplements, herbal supplements, review, scoping review

## Abstract

During the first year of the COVID-19 pandemic, the lack of cure and the intensity of the global spread raised a common awareness of health. The aim of this scoping review is to summarize dietary supplement use globally during first two years of the COVID-19 pandemic. A systematic search was conducted in December 2021 following PRISMA guidelines. PubMed, ERIC, and Scopus databases were searched, and 956 results were screened for eligibility. Fourteen cross-sectional studies from 11 countries and 3 continents were examined. All studies were large population surveys investigating healthy eating and supplement use during COVID-19. Vitamin C, vitamin D, zinc and multivitamins were the most widely reported, as well as natural/herbal products such as ginger and honey. The most common reason cited for supplements use was to strengthen immune system and to prevent infection of COVID-19. These studies reported that populations are relying on healthcare providers, family, friends, and social media to learn about supplement use. Future studies on the treatment of COVID-19 should include more evidence for supplement use.

## 1. Introduction

COVID-19 is still spreading worldwide, posing a serious threat to public health [1]. During the first year of the COVID-19 pandemic, the lack of cure and the intensity of the global spread raised a common awareness of health. The use of physical/social distancing, face masks, and eye protection were promoted as the most effective methods of reducing COVID-19 transmission and averting related chronic problems. Preventing transmission and decreasing the severity of the infection may also depend on lifestyle factors, such as nutrition, activity level, smoking, alcohol use, screen time, and sleep [2].

According to the United States Food and Drug Administration (FDA), dietary supplements are defined as products that are taken orally and contain a “dietary ingredient”. Vitamins, minerals, amino acids, herbs or botanicals that can be utilized to enhance the diet, are all examples of dietary ingredients [3]. Past literature discusses that supplements are generally consumed to maintain or improve overall health and meet nutritional needs [4,5].

SARS-CoV-2 (COVID-19) influences individual immunity, i.e., the severity of an infection is determined by one’s immune-competence; each person has a unique immune system, with everyday activities having a substantial influence on the immune system’s strength [6]. When various substances, for instance vitamins (including vitamins A, B6, B12, C, D, E, and folate) and elements like zinc, iron, selenium, magnesium, and copper) are inadequate, virus infections may cause immune system damage and a subsequent insufficient micronutrient reserve [7]. Distinct substances, such as essential fatty acids, linoleic acids, necessary amino acids, plus the vitamins and minerals described above, might increase immune response, particularly when immunity is conditioned by inadequacies, as in the case of viral infections [7]. While scientists are considering conventional pharmaceuticals, adjunctive treatments using vitamin therapy or even plant therapies are being considered. Many articles and review articles have been describing the possible role of supplementary vitamins and minerals in the treatment and prevention of COVID-19. For example, the efficacy of vitamin C, vitamin D and zinc with COVID-19 are being considered in clinical trials [8,9].

However according to National Institute of Health’s Office of Dietary Supplements, there is currently inadequate evidence to advocate the use of any vitamin, mineral, herb or other botanical, fatty acid, or other dietary supplement ingredient to prevent or treat COVID-19 [1]. Dietary supplements are also prohibited by law from being sold as a treatment, preventive, or cure for any disease; only pharmaceuticals are permitted to make such claims. Nonetheless, with the development of COVID-19, sales of dietary supplements advertised for immune health has escalated [1]. Along with these advertisements, the sales for supplements with the claim to boost immune health has also increased. According to GlobeNewswire, the report Global Immune Health Supplements Market Share, Trends, Analysis, and Forecasts, 2020–2030, examines important trends, business strategies, research and development activities, supply chain analysis, competitive landscape, and market composition analysis. The market for immune health supplements which was US $19 billion in 2020 is predicted to reach US$ 43.5 billion by 2031, with a compound annual growth rate of 7.7% [10].

The purpose of this review is to describe the perceptions, knowledge, and use of supplements among consumers during the COVID-19 pandemic, according to the literature from the first 2 years (2020–2021). These years in the emergence and management of COVID-19 are significant as there was still a lot unknown about the virus and vaccines were not available to most of the world.

## 2. Materials and Methods

A scoping search was conducted from August 2021 to December 2021. The point of this first scoping search was to review the extent of the literature and keywords related to healthy eating and dietary supplement use, perceptions, and surveys. Revision of keywords were made, and the following systematic search was conducted in December 2021, following PRISMA guidelines. Three databases (the PubMed, Scopus, and PsycINFO) were searched. The following keyword search was utilized: (“diet, healthy” OR “healthy eating” OR “food habits” OR “eating habits” OR “dietary habits” OR “dietary supplements” OR “dietary supplement*” OR “nutraceutical*” OR “multivitamin” OR “food supplement*” OR “herbal supplement*” OR “feeding behavior”) AND (“COVID” OR “COVID*” OR “Coronavirus” OR “pandemic”) AND (“attitude” OR “attitude*” OR “perception*” OR “opinion*” OR “survey*” OR “questionnaire*” OR “psychology” OR “belief”). The inclusion criteria were peer-reviewed journal articles, original research (excluding narrative reviews, books, systematic reviews) and those published in the English language, in years 2020–2021. Eligible studies were limited to adults 18 years and older. The exclusion criteria were studies published in languages other than English, those conducted with children, pregnant women or people suffering from any kind of health condition including obesity and eating disorders. Studies on specific populations for instance medical students, dietitians, physicians were also excluded.

## 3. Results

**Figure 1:**
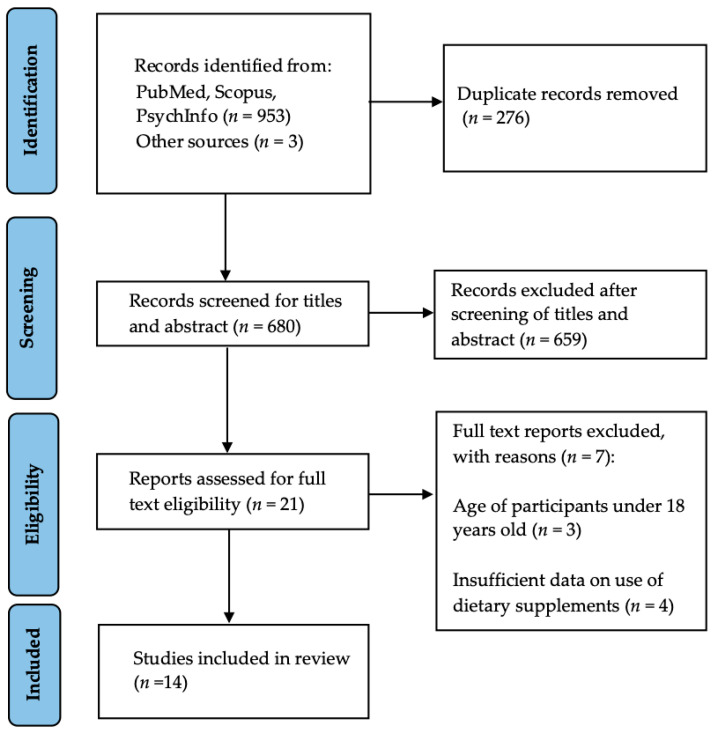
PRISMA 2020 flow diagram displaying the scoping review process.

A total 956 articles were identified, out of which 276 were duplicate articles. After removing duplicates, the remaining 680 articles were screened for title and abstracts of which 659 irrelevant articles were excluded. Twenty-one supplement related articles were further investigated for full text review. Seven articles were excluded due to following reasons: 3 articles due to age of the participants under 18 years [11,12,13], and 4 articles due to insufficient information regarding dietary supplement use [14,15,16,17]. Finally, 14 articles were included in this review (Figure 1).

### 3.1. Study Designs and Demographics

Table 1 gives an overview of the studies including the year study was published, author information, study type, location, number of participants, gender, mean age of participants and different themes included in this review. Most of the studies in this review relied on self-reported data from adult populations of different nations. All fourteen studies were cross-sectional. Most of these studies distributed their questionnaires through social media platforms such as Facebook, WhatsApp, WeChat, Twitter, Instagram, or through Gmail. A study from Ahmed et al. (2020) collected their data both in person and online through questionnaire [18]. Online surveys were available to participants for minimum of 2 weeks. Data collection for a study from Poland lasted nine months and included three cross-sectional surveys, each conducted at the end of a COVID-19 wave [19]. A study from Ali et al. (2021) does not mention the exact time frame of their survey conducted during lockdown [20]. Among the 14 studies, most participants were females, with the exception of one study from Bangladesh for which the majority of respondents stated they were male [18].

### 3.2. Dietary Supplements

There was total 12 papers reporting participants’ consumption of dietary supplements during COVID-19. In four papers from Asian countries, reported use of dietary supplements use during confinement ranged between 25–63% [18,20,26,31] (25.3%, *n* = 160), (37.7%, *n* = 772), (47%, *n* = 85) and (62.57%, *n* = 1224) respectively. In six papers from Middle East region countries, reported use of dietary supplements ranged between 15–71% [22,23,24,25,27,29] with (14.9%, *n* = 783), (25.5%, *n* = 145), (34%, *n* = 772), (36.35%, *n* = 381), (69.9%, *n* = 2076) and (71.5%, *n* = 309) respectively. In two papers from Europe, survey participants reported use was between 21–80% during confinement [19,30].

The most common types of dietary supplements reported were vitamin C, vitamin D, multivitamins, probiotics, omega-3, zinc, B complex vitamins; among these dietary supplements, the most frequently used supplements were vitamin C, vitamin D, multivitamins, and zinc with the range of population reported use (15–94%), (18–34%), (19–31%) and (3–18%) respectively. The most common reason cited for using dietary supplement during confinement was to strengthen immunity against COVID-19 to lower the risk of infection [18,21,23,25,26].

According to these studies, the most common factor influencing dietary supplements use during COVID-19 was gender. Females were more likely to report to consume dietary supplements compared to men [18,26,29,30]. Other factors like education level, age of participants, presence of any chronic disease/COVID-19 symptoms/infection, prior use of supplements before COVID-19, influenced use of supplements during COVID-19. Participants who have attained higher education were more likely to use supplements during COVID-19 [18,26]. Ahmed et al. (2020) reported, young participants, aged 19–29 years were using supplements during COVID-19 [18]. In contrast, Pérez-Rodrigo et al. (2021) and Lam et al. (2021) reported, participants who were middle aged 35–55 years and older aged more than 55 years were using supplements during COVID-19 [26,30]. Participants having any chronic disease or having COVID-19 related symptoms or infection were more likely to use supplements [18,26,27]. Participants who had used supplements before pandemic were more likely to use them during pandemic [26,27].

### 3.3. Natural Products/Herbal Products Use

There were six studies reporting the use of herbal/natural products during COVID-19 pandemic. The most common reason reported for using herbal supplements/natural products was to enhance/strengthen immunity against COVID-19 [21,25,26]. Two studies reported they were using these supplements during confinement to protect them from disease and to lower the risk of infection [18,23]. According to NIH National Center for Complementary and Integrative Health, herbal supplements, often known as botanicals, are nutritional supplements that include one or more herbs [32]. Three papers, two from Middle East and one from Asia, reported use of herbal supplements/natural products during confinement was between 50–64% [18,21,24] with (50% *n* = 1141), (57.6%, *n* = 704) and (64%, *n* = 955) respectively. Two studies, one from Asia and other from Middle East reported a decrease in use of herbal supplements /natural products comparing during confinement vs. before confinement. Mohsen et al. (2021) reported an insignificant change in the use of herbal/natural products. They reported 6.3% (*n* = 188) and 6% (*n* = 179) of respondents acknowledged to using herbal products before and during the pandemic [27]. Lam et al. (2021) reported from China, a significantly smaller proportion of respondents reported usage of traditional medicines during the pandemic (19.3%) compared to prior to the pandemic (28.6%). This may be due to lockdown policies preventing visits to see their practitioners [26].

The most common types of herbal supplements/ natural products reported were ginger, garlic, honey, turmeric, lemon, black seed, cinnamon, and anise. Among these products, ginger, honey, garlic, and turmeric (curcumin) were widely used among respondents with reported use between (32–56%), (30–46%), (10–34%) and (16–19%) respectively [18,21,24,25,27]. A study from China reported use of traditional Chinese herbal products by its participants during confinement: Lingzhi (Ganoderma Lucidum) (*n* = 7/278, 2.5%), Chrysanthemi Flos (*n* = 5/278, 1.8%), Isatidis Radix (*n* = 5/278, 1.8%), and Glycyrhizae Radix Et Rhizoma (*n* = 5/278, 1.8%) [26].

### 3.4. Supplement Knowledge, Attitudes and Beliefs

Alyami et al. (2020) reported beliefs about different nutrition and herbal supplements among its participants with majority of participants reporting use of vitamin C and garlic can reduce the chances of developing COVID-19 and can increase immunity [23]. A study by Alshammari et al. (2021) reported participants’ beliefs about vitamins that are essential for immunity and vitamins that can prevent from common cold/flu. Results indicated vitamin D scored higher followed by vitamin C in terms of immunity. However, when asked about common cold/flu prevention, vitamin C scored higher followed by vitamin D [22]. In contrast a study by Mohsen et al. (2021) reported, no significant change in participants’ belief, that vitamin C supplements can protect them from flu [27]. Ogundijo et al. (2021) asked its study participants from different ethnic groups in England about how important supplement use during COVID-19 would support their immune system. Overall, majority of participants taking supplements believed enhancing immunity was important. However, for 12.5% of study participants, supplement use was not important at all; the authors noted that and most of these respondents were from white ethnic group while the majority of those who responded it was greatest importance to use supplements were people from mixed or multiple ethnic groups [28]. A study from Pakistan by Ali et al. (2021) reported its participants (*n* = 1956) usage of vitamin-C and immune boosting supplements was related to lower level of knowledge about COVID-19. About 34–36% of overall participants were having poor knowledge related to these supplements use during COVID-19 [20].

### 3.5. Source of Information/Advice about Supplements and Natural/Herbal Products

There was a total of seven papers reporting participants’ source of information/advice about supplements and natural/herbal products use during COVID-19. The most common source of information/advice regarding dietary supplements use for participants were: family/friends, relatives/colleagues (36–41%) followed by social media/tv/internet/newspapers/advertisements (14–60%) and healthcare providers like doctors, physicians, dietitians, pharmacists, medical practitioners (13–43%) [18,19,22,23,26,27].

Mohsen et al. (2021) compared participants’ source of information/advice regarding supplements before and during pandemic. Majority of participants relied on medical prescriptions for supplement use during COVID-19 as compared to before COVID-19 58.9% vs. 54.9% respectively. However, findings showed that participants who used to follow the advice of dietitians, sports trainers, athletes, and friends had decreased by 1.5%, 4.4%, 0.6% and 1.4%, respectively, during pandemic compared to before pandemic. It was reported healthcare providers were the main source for information for participants concerns about supplements at both study periods, 61.4% before and 63.6% during, respectively. For example, in this study females reported using medical prescriptions (females: 76.7% before, 77.9% during) and seeking information about supplements from healthcare providers (females: 69.4% before, 63.9% during). In comparison, while a lower proportion of males reported using medical prescriptions than females (males: 40.5% before, 53.1% during) or seeking medical advice (males: 51.7% before, 63.2% during), the proportion of males seeking physicians regarding supplements increased. In addition, participants who reported getting information from other sources like friends, TV, sport trainers, supplement store salesperson, and mass media decreased by 0.7%, 0.1%, 3.4%, 0.8% and 0.6% during the COVID-19 pandemic, respectively [27]. In contrast, during the pandemic, the percentage of people who reported they got their information from reliable journals, books, and family members increased by 2.8%, 0.3%, and 0.7%, respectively [27]. A study from Jordon reported, participants use of herbal supplements during confinement and their preparation methods were based on information gathered from the internet or television shows [25].

## 4. Discussion

This scoping review investigated the consumer use and perspective on dietary supplement consumption during the first two years of COVID-19 among people from various geographical areas (Middle East, Europe, and Asia). According to the data from these articles, the use of supplements was more commonly reported by female participants. In this review it was reported that Vitamin C was most often recognized and used as an immune booster during the confinement period (15–94%).

The most common type of herbal/natural products used were ginger and honey. Ginger and honey have been used for centuries to address immunological health issues [33,34]. During the COVID-19 time period (2020–2021), most of the published studies concerning herbal and natural products were from Middle East. Herbal therapies were also reported in Asian countries, in particular China. Both of these regions have battled previous respiratory pandemics (H1N1 and MERS). These past experiences may have prompted multiple studies on consumer use during COVID-19.

Overall, studies reported that their participants know about the benefits of nutrients and have been self-managing using various supplements/herbal/food products. During the time of COVID-19 confinement, with lack of awareness and treatment options, people did try to rely on advice/information from health care providers like doctors and pharmacists regarding supplement use though some may not have been able to have medical visits during the lockdown periods. Supplements and natural products were reportedly used by educated populations as well as those who had less information about the COVID-19. Factors that influence the use of dietary supplements includes their wide availability. Supplements and natural products are widely available over the counter and via prescription, even online stores [35]. Studies on herbal supplement use during COVID-19 suggest consumers may have started their own gardens to use plants for preventive measures [36].

The role of vitamins and minerals have been evaluated for their efficacy in virus prevention and management for many years even prior to COVID pandemic. The consumer interest in vitamin C, D, and zinc from this current review mirrors the scientific discussions on vitamins and minerals prior to and during the COVID-19 pandemic. Multiple reviews on these vitamins and minerals show the mechanisms of how they can affect immunity but not enough conclusive evidence for using them as treatment [8,9,37,38,39]. For example, vitamin C is noted for strengthening immunity but there is not enough evidence on the role of high dosages of vitamin C to reduce viral symptoms for the common cold [37]. Vitamin D and zinc have been described to have many physiological benefits for the immune system, though the optimal recommended dose of vitamin D is still not clear [38]. Zinc has a wide range of physiological roles including it may prevent viral entry and block replication; however more study needs to be done for its efficacy and safety [39].

Herbal and natural products have been used in complementary and alternative medicines for thousands of years in Eastern countries. In their extensive systematic review, Fan et al., described 21 randomized controlled trials and 5 cohort studies, as well as other study designs that tested Chinese herbal medicines in conjunction with standard care for COVID-19. These studies have found supportive evidence that when used as a co-therapy, Chinese herbal medicines can decrease inflammation and may help reduce mortality [40].

Experts have now made statements supporting either vitamins and minerals or Chinese therapies to support treatment of COVID-19. In January 2021, 76 French experts released a united statement on their recognition of the role of vitamin D to decrease the severity of disease. They stated that the use of vitamin D by health care providers as a supplementary tool along with other methods is a promising and supported treatment [41]. To our knowledge there are no other government or expert reports recommending use of dietary supplements. For example, the regulatory institutions of governments assure the registration of safe products but do not endorse the claims on health. The position from the US FDA and health care guidance states that supplements are meant to help populations meet adequate nutrition but are not to be used as medicines [1]. The positions of European Union, Middle East, Spain and Bangladesh provide similar guidance as the US [42,43,44,45].

In 2022, WHO released a report after meeting with Chinese scientists, based on their study of randomized controlled trials on Chinese herbal therapies. The report concluded that the use of certain types of Chinese herbal supplements, also known within the category of complementary and alternative medicines, have been effective in reducing the severity of the novel COVID-19 [46]. A review of 25 guidelines found that only Chinese and South Korean guidelines were recommending Chinese herbal medicines [47].

A strength of our review is that we used the PRSIMA guidelines and provided a transparent and thorough search process. This is the first article to review the extent of preventative behavior through use and knowledge of dietary supplements. Our review has some limitations. As with any review, we are limited to those articles that have been published during the chosen critical timeframe and there was a lot of heterogeneity in how this topic was measured, from perceptions to use and level of importance. Our search included publications written only in English, which could have limited the results. These cross-sectional studies recruited participants through social media and internet and data were collected through online questionaries, which might have excluded some vulnerable populations, including those without internet or without reliable internet access. Most participants were recorded as female participants, which can lead to bias though it is known that females tend to respond to surveys and also use supplements [4].

## 5. Conclusions

All studies were large population surveys investigating healthy eating and dietary supplement use during COVID-19. These studies found that the most frequently used dietary supplements were vitamin C, vitamin D, multivitamins, and zinc. In terms of natural/herbal products use, ginger and honey were commonly reported. The most common reason regarding dietary supplements and natural/herbal products use was to strengthen immune system and to prevent infection of COVID-19. These studies reported that populations are relying on healthcare providers, friends and family, social media, and internet to learn about supplement use during COVID-19. Despite inadequate evidence regarding dietary supplement use to prevent or treat COVID-19, sales for these supplements are projected to increase. The reliance on supplements may have both short-term and long-term consequences on health. Future studies and interventions should aim to provide more adequate and accurate evidence for the use of dietary supplements in COVID-19 to the public.

## Figures and Tables

**Table 1:** Characteristics of cross-sectional studies from scoping search, by reported themes and dietary supplement use.

First Author	Location (Country) N	Male/ Female	Age (Years)	Themes	Types of Products Reported	Overall Used (%) & Often Used (%)
Ahmed et al. 2020 [18]	Asia (Bangladesh) 1222	750/466	18–82	Dietary supplements Natural/Herbal products Source of information/advice about supplements and natural/herbal products	Vitamins: B, C, & D Mineral: Zn Natural Products: tea (normal & herbal), black seed, ginger, honey, clove, cinnamon, garlic, lemon, black paper, cardamon, bay leaf & tulsi, Others: MV	Overall used (%) D.S (47%) Natural products (57.6%) Often used (%) Vitamins: B, C, D & MV (27%) Natural products: tea (71%), ginger (56.5%), black seed (32.8%), honey (30%) & clove (28.8%)
Ali et al. 2021 [20]	Asia (Pakistan) 1956	546/1410	18–65	Dietary supplements Knowledge, attitude, and beliefs	Vitamin: C Others: immune boosting supplements	Overall used (%) D.S (62.57%) Often used (%) Vitamin: C (41.4%)
AlNajrany et al. 2021 [21]	Middle East (Saudi Arabia) 1473	605/868	18+	Natural/Herbal products use	Vitamin: C Natural products: honey, lemon, ginger, black seed, garlic, turmeric, anise, myrrh, cinnamon & trigonella	Overall used (%) Natural products (64%) Often used (%) Vitamin: C (32.5%) Natural products: honey (46%), lemon (45%), ginger (36%), black seed (26%) & garlic (25.8%)
Alshammari et al. 2021 [22]	Middle East (Saudi Arabia) 575	247/328	18+	Dietary supplements Knowledge, attitude, and beliefs Source of information /advice about supplements and natural/herbal products	Vitamins Minerals Others: MV, creatine, amino acids, protein powder, sports drinks, weight loss products, caffeine & ginseng	Overall used (%) D.S (25.5%) Often used (%) Vitamins: (58.62%) Minerals: (24%) Others: MV (55%)
Alyami et al. 2020 [23]	Middle East (Saudi Arabia) 5258	2255/3002	18+	Dietary supplements Natural/Herbal products Knowledge, attitude, and beliefs Source of information/advice about supplements and natural/herbal products	Vitamin: C Others: royal jelly	Overall used (%) D.S or Natural products (14.9%) Often used (%) Vitamin: C (94.4%)
Bakhsh et al. 2021 [24]	Middle East (Saudi Arabia) 2255	802/1453	18+	Dietary supplements Natural/Herbal products	Vitamins: B, C, & D Minerals: Fe, Ca, Zn, & Mg Natural products: honey, lemon, ginger, orange, black seeds, turmeric & garlic Others: MV, omega 3	Overall used (%) D.S (34%) Natural products (50%) Often used (%) Vitamins: C (50%) & D (22%) Others: MV (26%) Natural products: honey (43%), lemon (40%) & ginger (32%)
Issa et al. 2021 [25]	Middle East (Jordon) 1048	256/792	18+	Dietary supplements Natural/Herbal products Source of information/advice about supplements and natural/herbal products	Vitamins: C, D & E Minerals: Zn, Mg & Se Natural products: ginger, mint, anise, green tea, sage, garlic, cinnamon, chamomile & cumin Others: MV	Overall used (%) D.S (36.35%) Natural products (37.98%) Often used (%) Vitamins: C (52.48%) & D (34.35%) Others: MV (31.20%) Natural products: ginger (49%), mint (45%), anise (41%), green tea (36%), sage (35%), garlic (34%), cinnamon (31%), chamomile (31%)
Lam et al. 2021 [26]	Asia (Hong Kong) 632	233/399	18+	Dietary supplements Natural/Herbal products Source of information /advice about supplements and natural/herbal products	Vitamins: B & C Natural products: Lingzhi, Chrysanthemi Flos, Isatidis Radix, Glycyrrhizae Radix Et Rhizoma & Yinqiao Jiedu Pian Others: fish oil & probiotics	Overall used (%) D.S (25.3%) Natural products (19.3%) Often used (%) Vitamin: C (24.8%) Natural product: Lingzhi (2.5%)
Mohsen et al. 2021 [27]	Middle East (Lebanon) 2966	1449/1522	18+	Dietary supplements Natural/Herbal products Knowledge, attitude, and beliefs Source of information/advice about supplements and natural/herbal products	Vitamins: A, B9, B12 C, D, & E Minerals: Ca, Fe, Mg, P & Zn Natural products: curcumin, ginger, oregano oil, coconut oil, aloe vera, anise, cumin, chamomile, honey, green tea, and garlic Others: MV, antioxidants	Overall used (%) D.S (69.9%) Natural products (6%) Often used (%) Vitamins: C (42%), D (41%) & E (17.5%) Minerals: Zn (29%)
Ogundijo et al. 2021 [28]	Europe (England) 792	274/506	18–91	Supplements knowledge, attitude, and beliefs	n/a	n/a
Özenoğlu et al. 2021 [29]	Middle East (Turkey) 432	120/312	18+	Dietary supplements	Vitamins: B complex, C & D Minerals: Fe & Zn Others: MV, omega 3 & probiotics	Overall used (%) D.S (71.5%) Often used (%) Vitamins: C (25.9%) & D (28.7%) Others: MV (19%) Natural products: ginger & turmeric (19%)
Pérez-Rodrigo et al. 2021 [30]	Europe (Spain) 1036	301/735	18+	Dietary supplements	Vitamins: A, B9, B12, B complex, C, D & E Minerals: Ca, Mg, Se, I & Zn Others: MV, brewers yeast, fiber, omega-3 & probiotics	Overall used (%) D.S (21%) Often used (%) Vitamins: C (22.2%) & D (25.8%) Others: MV (27%), brewer yeast (16.8%), fiber (16.8%), omega 3 (15.9%) & probiotics (12.4%)
Puścion-Jakubik et al. 2021 [19]	Europe (Poland) 935	181/754	18+	Dietary supplements Knowledge, attitude, and beliefs Source of information /advice about supplements and natural/herbal products	Vitamin: D Mineral: Zn Others: prebiotics & probiotics	Overall used (%) D.S (80%) Often used (%) Vitamin: D (37.6%)
Zhao et al. 2020 [31]	Asia (China) 1938	665/1273	18–80	Dietary supplements	Vitamin: C Others: probiotics & Chinese herbs	Overall used (%) D.S (37.7%) Often used (%) Vitamin: C (18.2%)

D.S = Dietary Supplements, MV = Multi Vitamins, Zn = Zinc, P = Phosphorus, Ca = Calcium, Se = Selenium, Mg = Magnesium, Fe = Iron, I = Iodine, n/a = not applicable, N = number of study participants.

## Data Availability

Not applicable.
